# Factors Predicting Malignant Occurrence and Polyp Recurrence after the Endoscopic Resection of Large Colorectal Polyps: A Single Center Experience

**DOI:** 10.3390/medicina58101440

**Published:** 2022-10-13

**Authors:** Olga Mandic, Igor Jovanovic, Mirjana Cvetkovic, Jasmina Maksimovic, Tijana Radonjic, Maja Popovic, Novica Nikolic, Marija Brankovic

**Affiliations:** 1 Department of Gastroenterology and Hepatology, University Hospital Medical Center Bezanijska Kosa, 11000 Belgrade, Serbia; 2 Department of Radiology, University Hospital Medical Center Bezanijska Kosa, 11000 Belgrade, Serbia; 3 Department of Anesthesiology and Reanimation, University Hospital Medical Center Bezanijska Kosa, 11000 Belgrade, Serbia; 4 Faculty of Medicine, University of Belgrade, 11000 Belgrade, Serbia

**Keywords:** colorectal polyp, endoscopic mucosal resection, malignancy, recurrence

## Abstract

*Background:* The aim of this study was to identify risk factors contributing to the malignancy of colorectal polyps, as well as risk factors for recurrence after the successful endoscopic mucosal resection of large colorectal polyps in a referral center. *Materials and Methods*: This retrospective cohort study was performed in patients diagnosed with large (≥20 mm diameter) colorectal polyps and treated in the period from January 2014 to December 2019 at the University Hospital Medical Center Bezanijska Kosa, Belgrade, Serbia. Based on the endoscopic evaluation and classification of polyps, the following procedures were performed: en bloc resection, piecemeal resection or surgical treatment. *Results*: A total of 472 patients with large colorectal polyps were included in the study. The majority of the study population were male (62.9%), with a mean age of 65.7 ± 10.8 years. The majority of patients had one polyp (73.7%) less than 40 mm in size (74.6%) sessile morphology (46.4%), type IIA polyps (88.2%) or polyps localized in the descending colon (52.5%). The accessibility of the polyp was complicated in 17.4% of patients. En bloc resection was successfully performed in 61.0% of the patients, while the rate of piecemeal resection was 26.1%. Due to incomplete endoscopic resection, surgery was performed in 5.1% of the patients, while 7.8% of the patients were referred to surgery directly. Hematochezia (*p* = 0.001), type IIB polyps (*p* < 0.001) and complicated polyp accessibility (*p* = 0.002) were significant independent predictors of carcinoma presence in a multivariate logistic regression analysis. Out of the 472 patients enrolled in the study, 364 were followed after endoscopic resection for colorectal polyp recurrence, which was observed in 30 patients (8.2%) during follow-up. Piecemeal resection (*p* = 0.048) and incomplete resection success (*p* = 0.013) were significant independent predictors of polyp recurrence in the multivariate logistic regression analysis. *Conclusions:* Whenever an endoscopist encounters a complex colorectal lesion (i.e., a polyp with complicated accessibility), polyp size > 40 mm, the Laterally Spreading Tumor nongranular (LST-NG) morphological type, type IIB polyps or the presence of hematochezia, malignancy risk should be considered before making the decision to either resect, refer to an advanced endoscopist or perform surgery.

## 1. Introduction

Colorectal cancer is the third most common cancer, as well as the second leading cause in terms of cancer-related deaths worldwide [1]. With 5989 new cases and 3356 registered deaths from colorectal cancer in 2020, data from the International Agency for Research on Cancer (IARC) put Serbia in fifth place in the world in terms of the number of deaths caused by colorectal cancer [2]. Although every colorectal neoplasia has malignant potential, polyps classified as large colon polyps (≥2 cm) carry an even greater risk for carcinoma development [3,4,5,6,7]. In order to avoid unnecessary surgery, many large colon polyps are removed endoscopically, making endoscopic resection the most effective strategy for the prevention and decline of colorectal cancer mortality, morbidity and cost [8]. Data from the literature show that up to 11% of colorectal polyps that are endoscopically removed are already malignant [9]. In addition, it was shown in one study that 15% of local recurrences of adenoma occurred after an endoscopic resection procedure and about 88% of recurrences were detected after the first follow-up colonoscopy [10]. In most cases, these local recurrences can be managed endoscopically [11]; however, rigorous surveillance is needed in order to detect them early on. Therefore, the recognition of factors contributing to malignancy as well as local recurrence predictive factors may enhance the prevention of this disease, primarily through the stratification of patients according to their individual risk profile and polyp-related characteristics.

The aim of this study was to identify risk factors contributing to the malignancy of colorectal polyps, as well as risk factors for recurrence after the successful endoscopic mucosal resection of large colorectal polyps in a referral center.

## 2. Materials and Methods

This retrospective cohort study was performed on patients diagnosed with large (≥20 mm diameter) colorectal polyps and treated in the period from January 2014 to December 2019 by an expert endoscopist in the referral University Hospital Medical Center Bezanijska Kosa, Belgrade, Serbia. Patients who were positive for fecal occult blood test (FOBT) as a part of a National Screening Program were also assessed in the study. Basic demographic and clinical data were obtained for all patients (age, gender, comorbidities, previous history of carcinoma and indications for colonoscopy). In addition, polyp characteristics, including number, size, morphology, pit pattern classification and location, were documented. The accessibility of polyps was defined as complicated if the polyps were located in difficult sites, such as the appendiceal orifice, ileocecal valve or anorectal junction, or if they were located behind haustral folds. The Paris Classification System for Superficial Neoplastic Lesions in the Digestive Tract and the classification for Laterally Spreading Tumors (LST) were used to define polyp morphology. The Japan NBI Expert Team (JNET) Classification was used to describe the characteristics of the mucosal surface. The inclusion criteria were patients ≥ 18 years and the presence of one or more polyps, over 20 mm in diameter. The absence of a pathological evaluation of the polyp was the exclusion criterion.

A colonoscopy was performed using an Olympus CF-H170L video colonoscope. Based on the endoscopic evaluation and classification of polyps, the following procedures were performed: en bloc resection, piecemeal resection or surgical treatment due to incomplete endoscopic mucosal resection or the presence of a likely malignant alteration. While performing the endoscopic mucosal resection, a saline solution of adrenaline at a concentration of 1:10,000 was injected into the base of the polypoid change until an adequate elevation of the change was achieved. After the elevation, the polypoid change was removed with a hexagonal loop, en bloc or piece by piece, with the help of an electrocoagulation unit. The evaluation of the endoscopic resectability was based on the presence of a “lifting” sign after the submucosal injection.

The study was approved by the Institutional Research Ethic Committee and informed consent was obtained from all patients.

### Statistical Analysis

Numerical data were presented as mean values with standard deviation for numerical variables, or as absolute numbers with percentages for categorical variables. Differences according to the presence of carcinoma or polyp recurrence in patients’ sociodemographic and polyp characteristics and were analyzed using Student’s t and Chi-square tests for the numerical and categorical data, respectively. Univariate and multivariate logistic regression models were used to assess the predictors of carcinoma and polyp recurrence (as dependent variables). In all analyses, the significance level was set at 0.05. Statistical analysis was performed using the IBM SPSS statistical software (SPSS for Windows, release 25.0, SPSS, Chicago, IL, USA).

## 3. Results

A total of 472 patients with endoscopically resected colorectal polyps were included in the study. The majority of the study population were male (62.9%) with a mean age of 65.7 ± 10.8 years. The patient characteristics are summarized in Table 1.

The majority of patients had one polyp (73.7%) less than 40 mm in size (74.6%), sessile morphology (46.4%), IIA type (88.2%) and localized in the descending colon (52.5%). The accessibility of the polyp was complicated in 17.4% of patients (Table 2).

En bloc resection was successfully performed in 61.0% of the patients, while the rate of piecemeal resections performed was 26.1%. Due to incomplete endoscopic resection, surgery was performed in 5.1% of the patients, while 7.8% of the patients were referred to surgery directly.

The characteristics of the study population and polyps according to carcinoma presence are presented in Table 3. Patients with hematochezia, polyps ≥ 40 mm in size, LST-NG morphology, type IIB, localized in the cecum and polyps with complicated accessibility were more frequently diagnosed with carcinoma, while patients with an FOBT-positive indication, pedunculated polyps and polyps localized in the transverse colon were diagnosed with carcinoma less frequently (Table 3).

The results from univariate and multivariate logistic regression analyses with carcinoma as the dependent variable are presented in Table 4. Hematochezia (*p* = 0.001), polyp size over 40 mm (*p* < 0.001), morphological type LST-NG (*p* = 0.036), type IIB polyps (*p* < 0.001) and complicated accessibility (*p* < 0.001) were significant predictors of carcinoma presence in the univariate logistic regression analysis. Hematochezia (*p* = 0.001), type IIB polyps (*p* < 0.001) and complicated accessibility (*p* = 0.002) were significant independent predictors of carcinoma presence in the multivariate logistic regression analysis (Table 4).

Out of the 472 patients enrolled in the study, 364 were followed after endoscopic resection for colorectal polyp recurrence, which was observed in 30 patients (8.2%) during follow-up. Patients who had polyp recurrence more often had previous surgery for colorectal carcinoma, had a single polyp over 40 mm in size, morphologically sessile polyps, LST-NG and LST-G, type IIB, or had polyps localized in the rectum, descending colon or ascending colon. In addition, patients who had polyp recurrence were more often treated via piecemeal resection with incomplete resection success, had complicated accessibility, had a tubulovillous adenoma according to the pathological diagnosis, or were less likely to have a clip placed than patients who did not have polyp recurrence (Table 5).

The results from univariate and multivariate logistic regression analyses with polyp recurrence as the dependent variable are presented in Table 6. Single polyp (*p* = 0.028), size over 40 mm (*p* < 0.001), morphological types LST-G (*p* = 0.003), LST-NG (*p* < 0.001), complicated accessibility (*p* = 0.001), piecemeal resection (*p* < 0.001), incomplete resection success (*p* < 0.001) and polyps localized in rectum (*p* = 0.003) and ascending colon (*p* = 0.014) were significant predictors of polyp recurrence, while sessile polyp morphology (*p* = 0.001) and polyps localized in the descending colon (*p* < 0.001) represented protective factors for polyp recurrence in the univariate logistic regression analysis. Piecemeal resection (*p* = 0.048) and incomplete resection success (*p* = 0.013) were significant independent predictors of polyp recurrence in the multivariate logistic regression analysis (Table 6).

## 4. Discussion

The results of our study showed that hematochezia, type IIB polyps, and complicated accessibility were significant independent predictors of carcinoma development, while piecemeal resection and incomplete resection success were significant independent predictors of polyp recurrence in a multivariate logistic regression analysis. Protective factors for polyp recurrence were sessile polyp morphology and polyps localized in the descending colon.

In 2021. Cazacu et al. [12] performed a retrospective study of patients with colonoscopic polypectomy during a 13-year period; out of 905 patients with colonoscopic polyps, pathological examination showed polyps with malignant cells in 109 patients. The results of this study showed that the frequency of male patients with malignant polyps was similar to the group of patients with benign polyps, and that the mean age of the patients was 62.6 years. In addition, the prevalence of the malignant polyps in male patients varies across the literature, with 51 to 88% of male patients having carcinoma and a mean age between 60 and 73 years [13,14,15,16,17,18,19]. These results are in agreement with our study results, where 57.4% of male patients with mean age of 66.8 years had malignant polyps upon pathological examination. No statistical significance was found between gender or age and carcinoma diagnosis in our study; however, in the study by Cazacu et al. [12], older patients (≥65 years) had a higher rate of carcinoma in comparison to the younger population.

In the study by Cazacu et al., statistical significance was reported for the mean diameter between benign and malignant polyps [12], which is similar to our study results, where patients with polyps ≥ 40 mm in size were more frequently diagnosed with carcinoma. Our study results showed that most of the pathologically examined polyps were sessile, LST-NG and LST-G in the group of patients with carcinoma. The predominance of sessile polyps was found in other studies [12,17], in line with our study results. However, a meta-analysis conducted by Hassan et al. [20] revealed the predominance of pedunculated polyps in patients with colorectal polyps; in our study, only 9.3% of patients had pedunculated polyps. Statistical significance was found for the frequency of pedunculated polyps between the groups of patients with and without carcinoma, where patients with pedunculated polyps were less frequently diagnosed with carcinoma. Traditionally, pedunculated malignant polyps are considered to prevent recurrent disease and have a better prognosis in comparison to sessile lesions [21,22].

The results reported by Cazacu et al. [12] showed that the rate of malignancy in colorectal polyps was higher in patients with two or more polyps, patients with polyps larger than 10 mm in size, in polyp types 0-Ip and 0-Isp (according to JNET classification), in lateral spreading lesions (as compared with flat and sessile lesions) and polyps localized on the left-side, as well as in the rectum. Our study results were similar, where hematochezia, polyp size over 40 mm, morphological type LST-NG, type IIB polyps (according to JNET classification) and complicated accessibility were significant predictors of carcinoma development in the univariate logistic regression analysis. In addition, in the multivariate logistic regression analysis, hematochezia, polyp type IIB (according to JNET classification) and complicated accessibility were significant independent predictors of carcinoma development.

In a study conducted by Nanda et al. [23], lesions considered to be challenging for the technical success of EMR procedure were discussed. The results of their study showed that polyps localized at the ileocecal valve were more challenging to position and access for resection, making resection more complicated as well as the duration of the procedure longer. Moss et al. [24] showed that 7.9% of their study cohort had polyps that were difficult to reach and were located in a difficult position for resection. In addition, Moss et al. [24] assessed risk factors for EMR failure, where the independent predictors in a multivariate regression analysis were polyps located in the ileocecal valve and a difficult position of the polyp. According to Sidhu et al. [25], polyp access may be considered difficult depending on the location of the lesion or whether a stable position is unable to be maintained by the endoscopist when performing EMR. In addition, the assessment of polyp access may not be mentioned specifically by the referring endoscopist. However, Sidhu et al. [25] found that short-term and procedural outcomes were significantly correlated with the size, morphology, site and access (SMSA) score level, even with lesions marked as “easy access”. The results of our study showed that complicated accessibility represented a significant predictor of carcinoma development in both univariate and multivariate regression analyses. The complicated accessibility of polyps could affect their early and accurate morphological evaluation, thus prolonging timely endoscopic mucosal resection and increasing the chance of carcinoma development. Future studies concerning the relationship between polyp accessibility and its malignant potential are needed in order to facilitate early identification, better management and the provision of future directions for how to achieve the best optimal outcome for patients with large polyps.

In the case of malignant colorectal polyps, a consensus was reached concerning several carcinogenic factors contributing to colorectal polyps; however, it remains unclear which factors contribute to the recurrence of colorectal polyps and whether these factors are similar to the factors that contribute to carcinogenesis [9,26,27,28]. In 2017, the European Society of Gastrointestinal Endoscopy (ESGE) Clinical Guideline by Ferlitsch et al. [29] recommended that features related to the recurrence of polyps should include polyps over 40 mm in size, polyps localized on the ileocecal valve, prior failed attempts at resection as well as size, morphology, site and access (SMSA) level 4. Apart from the ESGE guidelines, SMSA score was shown to predict the outcomes of endoscopic mucosal resection in the colon [25]. In a study by Chlebowski et al., the rate of polyp recurrence was shown to be higher in male patients than in female patients [30]. In another study conducted by Saiken A et al. [31], patients over 60 years old had higher rates of colorectal recurrence in comparison to younger patients. Gender and age were not found to be predictive risk factors of colorectal polyp recurrence in the study by Chaoui et al. [32], which is in agreement with our study results. In terms of polyp characteristics, several factors can contribute to local recurrence risk. A greater tendency of recurrence was shown for colorectal polyps located in the proximal and ascending colon in a study by Atkin et al. [33]. The results of our study showed that polyps localized in the rectum and ascending colon were significant predictors of polyp recurrence, while polyps localized in the descending colon represented protective factors for polyp recurrence in the univariate logistic regression analysis. In addition, a potential risk factor for the recurrence of adenoma is the size of colorectal polyp; the results of a meta-analysis conducted in 2016 showed that endoscopic recurrence occurred in 13.8% of patients with large colorectal polyps (≥20 mm in size) [34]. In the study conducted by Zhan et al., polyp size was significantly associated with the risk for polyp recurrence in a multivariable regression analysis [35]. Other studies have also demonstrated that a predictor of polyp recurrence is large polyp size [24,36,37]; this is in agreement with our study results, where polyp size over 40 mm was a significant predictor of polyp recurrence.

A growing number of studies have shown that although it is considered to be a safe and effective endoscopic treatment for large colorectal polyps, recurrence after EMR has been a point of contention since this technique emerged [11,34,38]. A meta-analysis conducted by Belderbos et al. showed that after a piecemeal resection, the recurrence rate of colorectal polyps was higher than after en bloc resection [10]. Additionally, the results of a study conducted by Moss et al. [11] showed that en bloc resection was associated with lower rates of recurrence than piecemeal resection. Our study results are in agreement with the abovementioned findings, where piecemeal resection was shown to be a significant predictor of polyp recurrence in both univariate and multivariate logistic regression analyses. In the last two decades, management strategies for colonic neoplasia have evolved considerably, leading to a paradigm shift from surgery to endoscopic resection. Due to an improved understanding of the pathophysiology of polyps, as well as ongoing advancements in technology, such as the development of novel endoscopic techniques and tools, clinical predictors of malignant colonic neoplasia have been well studied, yet factors that contribute to improved clinical decision making are still lacking. Most large (≥20 mm diameter) colorectal polyps can be removed with advanced endoscopist techniques; however, these procedures require a center with the proper equipment and trained staff, specifically an endoscopic expert in the field. Whenever an endoscopist encounters a complex colorectal lesion (with complicated accessibility), a polyp size > 40 mm, the morphological type LST-NG, a type IIB polyp or the presence of hematochezia, malignancy risk should be considered before making the decision to either resect, refer the patient to an advanced endoscopist or perform surgery.

Our study has several limitations. Despite the prospective enrollment of the patients, the data were collected and analyzed retrospectively. Second, the performed procedures were supervised by experienced endoscopists, thus making the results non-generalizable to centers where these procedures are performed by less advanced endoscopists. Furthermore, considering the small number of patients with malignancy and the recurrence of disease, as well as incomplete patient medical histories, any additional risk factors might not have been detected. Therefore, future prospective studies with larger patient cohorts using a longer surveillance period are needed in order to validate our findings.

## 5. Conclusions

In lesions without overt evidence of colorectal carcinoma, an evidence-based risk estimation approach may be used to choose the correct resection modality for large colorectal polyps. In order to optimize clinical outcomes and minimize the rate of adverse events, endoscopic resection should be performed based on patient-specific characteristics, local availability and expertise.

## Figures and Tables

**Table 1:** Characteristics of the study population.

Variable	n (%)
**Gender**	
Male	297 (62.9)
Female	175 (37.1)
**Age, mean ± sd**	65.7 ± 10.8
**Comorbidities, yes**	325 (68.9)
**Previous history of carcinoma, yes**	28 (5.9)
**Indication for colonoscopy**	
Symptoms	131 (27.9)
Fecal occult blood test positive	113 (24.0)
Hematochezia	98 (20.9)
Family history	45 (9.6)
Polyp surveillance	33 (7.0)
Anemia	28 (6.0)
Combination of two or more indications	22 (4.7)

**Table 2:** Polyp characteristics of the study population. Abbreviations: LST-NG, Laterally Spreading Tumor nongranular; LST-G, Laterally Spreading Tumor granular).

Polyps	n (%)
**Number**	
One	240 (50.8)
More than one	232 (49.2)
**Size**	
<40 mm	352 (74.6)
≥40 mm	120 (25.4)
**Morphology**	
Sessile	219 (46.4)
LST-NG	100 (21.2)
LST-G	69 (14.6)
Pedunculated	44 (9.3)
Flat	39 (8.3)
Hyperplastic	1 (0.2)
Pit pattern classification according to JNET *	
IIA	411 (88.2)
IIB	55 (11.8)
**Location**	
Cecum	40 (8.5)
Ascending colon	57 (12.1)
Transverse colon	37 (7.8)
Descending colon	248 (52.5)
Rectum	90 (19.1)
**Accessibility**	
Non-complicated	366 (82.6)
Complicated	77 (17.4)

* JNET—Japan NBI Expert Team Classification.

**Table 3:** Characteristics of the study population and polyps according to carcinoma presence.

Variable	Carcinoma		*p*
	No (n = 404)	Yes (n = 68)	
**Characteristics of the study population, n%**			
**Gender**			
Male	258 (63.9)	39 (57.4)	0.304
Female	146 (36.1)	29 (42.6)	
**Age, mean ± sd**	65.5 ± 10.8	66.8 ± 10.6	0.357
**Comorbidities, yes**	273 (67.6)	52 (76.5)	0.143
**Indication for colonoscopy**			
Symptoms	114 (28.2)	17 (25.0)	0.584
Fecal occult blood test positive	104 (25.7)	9 (13.2)	**0.025**
Hematochezia	73 (18.1)	25 (36.8)	**<0.001**
Family history	42 (10.4)	3 (4.4)	0.120
Polyp surveillance	29 (7.2)	4 (5.9)	0.698
Anemia	24 (5.9)	4 (5.9)	0.985
Combination of two or more indications	16 (4.0)	6 (8.8)	0.078
**Polyp characteristics, n%**			
**Number**			
One	206 (51.0)	34 (50.0)	0.880
More than one	198 (49.0)	34 (50.0)	
**Size**			
<40 mm	321 (79.5)	31 (45.6)	**<0.001**
≥40 mm	83 (20.5)	37 (54.4)	
**Morphology**			
Sessile	190 (47.0)	29 (42.6)	0.503
LST-NG	79 (19.6)	21 (30.9)	0.034
LST-G	57 (14.1)	12 (17.6)	0.445
Pedunculated	44 (10.9)	0 (0.0)	0.004
Flat	33 (8.2)	6 (8.8)	0.856
Hyperplastic	1 (0.2)	0 (0.0)	0.681
Pit pattern classification according to JNET *			
IIA	379 (95.2)	32 (47.1)	**<0.001**
IIB	19 (4.8)	36 (52.9)	
**Location**			
Cecum	30 (7.4)	10 (14.7)	0.046
Ascending colon	53 (13.1)	4 (5.9)	0.090
Transverse colon	36 (8.9)	1 (1.5)	0.035
Descending colon	211 (52.2)	37 (54.4)	0.739
Rectum	74 (18.3)	16 (23.5)	0.311
**Accessibility**			0.311
Non-complicated	334 (85.9)	32 (59.3)	**<0.001**
Complicated	55 (14.1)	22 (40.7)	

* JNET—Japan NBI Expert Team Classification. Abbreviations: LST-NG, Laterally Spreading Tumor nongranular; LST-G, Laterally Spreading Tumor granular).

**Table 4:** Univariate and multivariate logistic regression analyses with carcinoma presence as the dependent variable.

Variable	Univariate			Multivariate		
	*p*	OR	95%CI for OR	*p*	OR	95%CI for OR
Hematochezia	0.001	2.636	1.514–4.589	0.001	3.173	1.578–6.377
Size of polyps	<0.001	4.616	2.704–7.880			
LST-NG	0.036	1.838	1.039–3.251			
Pit pattern classification according to JNET *	<0.001	22.441	11.568–43.532	<0.001	12.505	5.710–27.386
Accessibility of polyp	<0.001	4.175	2.261–7.708	0.002	3.020	1.478–6.169

* JNET—Japan NBI Expert Team Classification. Abbreviations: LST-NG, Laterally Spreading Tumor nongranular).

**Table 5:** Characteristics of the study population and polyps according to polyp recurrence.

Variable	Polyp Recurrence		*p*
	No (n = 334)	Yes (n = 30)	
**Characteristics of the study population, n%**			
**Gender**			
Male	219 (65.6)	20 (66.7)	0.903
Female	115 (34.4)	10 (33.3)	
**Age, mean ± sd**	65.40 ± 10.33	68.60 ± 9.82	0.104
**Indication for colonoscopy**			
Symptoms	88 (26.3)	9 (30.0)	0.655
Fecal occult blood test positive	87 (26.0)	5 (16.7)	0.257
Hematochezia	60 (18.0)	5 (16.7)	0.859
Family history	33 (9.9)	3 (10.0)	0.983
Polyp surveillance	24 (7.2)	6 (20.0)	**0.014**
Anemia	23 (6.9)	1 (3.3)	0.453
Combination of two or more indications	18 (5.4)	1 (3.3)	0.628
**Polyp characteristics, n%**			
**Number**			
One	162 (48.5)	21 (70.0)	**0.024**
More than one	172 (51.5)	9 (30.0)	
**Size**			
<40 mm	246 (73.7)	12 (40.0)	**<0.001**
≥40 mm	88 (26.3)	18 (60.0)	
**Morphology**			
Sessile	215 (49.2)	4 (11.4)	**<0.001**
LST-NG	83 (19.0)	17 (48.6)	**<0.001**
LST-G	58 (13.3)	11 (31.4)	**0.003**
Pedunculated	43 (9.8)	1 (2.9)	0.172
Flat	37 (8.5)	2 (5.7)	0.569
Hyperplastic	1 (0.2)	0 (0.0)	0.777
Pit pattern classification according to JNET *			
IIA	384 (89.1)	27 (77.1)	**0.035**
IIB	47 (10.9)	8 (22.9)	
**Location**			
Cecum	77 (17.6)	13 (37.1)	**0.005**
Ascending colon	239 (54.7)	9 (25.7)	**0.001**
Transverse colon	36 (8.2)	1 (2.9)	0.254
Descending colon	49 (11.2)	8 (22.9)	**0.042**
Rectum	36 (8.2)	4 (11.4)	0.514
**Accessibility**			
Non-complicated	346 (84.4)	20 (60.6)	**0.001**
Complicated	64 (15.6)	13 (39.4)	
**Type of treatment**			
En bloc resection	205 (61.4)	2 (6.7)	**<0.001**
Piecemeal resection	79 (23.7)	25 (83.3)	
Surgery due to incomplete endoscopic resection	19 (5.7)	2 (6.7)	
Patients referred directly to surgery	31 (9.3)	1 (3.3)	
**Resection success**			
Complete	346 (84.4)	20 (60.6)	**0.001**
Incomplete	64 (15.6)	13 (39.4)	
**Pathological diagnosis**			
Tubular adenoma	105 (31.4)	7 (23.3)	0.357
Tubulovillous adenoma	166 (49.7)	21 (70.0)	**0.033**
Hyperplastic	6 (1.8)	0 (0.0)	0.459
Villous adenoma	2 (0.6)	0 (0.0)	0.671
Peutz–Jeghers	1 (0.3)	0 (0.0)	0.764
Intramucosal carcinoma (Tis)	15 (4.5)	1 (3.3)	0.767
Submucosal carcinoma (T1)	34 (10.2)	0 (0.0)	0.066
Carcinoma T2 or more	7 (2.1)	1 (3.3)	0.658
**Clip placement, yes**	208 (62.3)	10 (33.3)	**0.002**

*JNET—Japan NBI Expert Team Classification. Abbreviations: LST-NG, Laterally Spreading Tumor nongranular; LST-G, Laterally Spreading Tumor granular).

**Table 6:** Univariate and multivariate logistic regression analyses with polyp recurrence as the dependent variable. Abbreviations: LST-NG, Laterally Spreading Tumor nongranular; LST-G, Laterally Spreading Tumor granular).

Variable	Univariate			Multivariate		
	*p*	OR	95%CI for OR	*p*	OR	95%CI for OR
Number of polyps	0.028	0.404	0.180–0.907			
Size of polyps	<0.001	4.193	1.942–9.056			
LST-G	0.003	3.367	1.510–7.510			
LST-NG	<0.001	4.302	1.999–9.257			
Sessile	<0.001	0.076	0.018–0.323			
Accessibility of polyp	0.001	3.925	1.782–8.642			
Piecemeal resection	<0.001	16.139	5.980–43.556	0.048	3.870	1.011–14.819
Resection success	<0.001	17.098	5.808–50.332	0.013	6.363	1.478–27.385
Location (rectum)	0.003	3.240	1.479–7.096			
Location (descending colon)	<0.001	0.159	0.059–0.426			
Location (ascending colon)	0.014	3.010	1.249–7.257			

## Data Availability

The data that support the findings of this study are available on request from the corresponding author.
